# New perspectives in behavioural development: adaptive shaping of behaviour over a lifetime?

**DOI:** 10.1186/1742-9994-12-S1-S1

**Published:** 2015-08-24

**Authors:** Fritz Trillmich, Anja Günther, Caroline Müller, Klaus Reinhold, Norbert Sachser

**Affiliations:** 1 Animal Behaviour, Bielefeld University, Morgenbreede 45, 33615 Bielefeld, Germany; 2 Chemical Ecology, Bielefeld University, Universitätsstraße 25, 33615 Bielefeld, Germany; 3 Evolutionary Biology, Bielefeld University, Morgenbreede 45, 33615 Bielefeld, Germany; 4 Behavioural Biology, University of Münster, Badestraße 13, 48149 Münster, Germany

## Summary

The last years have seen an explosion of publications on consistent, i.e. repeatable, behavioural differences among individuals and their potential adaptive significance and evolutionary origin. Stable, repeatable traits imply reduced plasticity and this is particularly interesting in the case of behaviour, which is the most flexible trait of an animal in its interaction with the biotic and abiotic environment. While the importance of consistent individual differences has been widely acknowledged, the lifetime development of these differences and their potentially adaptive or maladaptive consequences for individuals only recently came into the focus of research [1,2]. As a result, widespread evidence for the existence of changes in behaviour and behavioural plasticity over the lifetime of individuals begins to accumulate [3]. This has raised novel questions concerning the overwhelming importance of ontogenetic and trans-generational influences on the development and fitness of behavioural phenotypes and the mechanisms influencing changes during an individual's lifetime.

Much theoretical work has been published on the importance of and limits to individual plasticity, mostly in the framework of the reaction norm. Less empirical work has actually studied, how the reaction norm comes about in an individual's lifetime and how or whether it may change over the lifetime [4]. This raises important questions about the adaptivity of the phenotypic plasticity of behaviour and the question when within a lifetime such plasticity might be particularly relevant. Do particular windows of plasticity exist, and if so why are they where they are within the lifetime of an organism? The potential lifetime plasticity also raises the question about the evolution of the reaction norm and, more recently, about the evolution of its plasticity, questions that so far have received but little attention. In these fields of investigation theory has moved far ahead of empirical studies. Often, if not always, individuals face uncertain environmental conditions throughout their life, forcing them to adapt plastically in order to maximise fitness. Under such uncertain conditions we expect natural selection to favour plasticity. However, plastic responses to the environment can only be adaptive, if informative environmental cues exist that allow prediction of future conditions with reasonably high probability [5]. Depending on the cues available and the ontogenetic stage of an individual we expect differential effects of such predictive cues: Early in life highly informative cues, whether derived from maternal influences or perceived directly, may potentially act in an organisational manner, whereas less informative cues or cues perceived at a later age may lead to more immediate plasticity (see [4] for a definition of different types of plasticity). This may lead to developmental influences on plasticity, i.e. early environments may particularly influence the reaction norm. This idea of plasticity of the reaction norm as a trait that develops and thereby influences the reaction norm expressed during the lifetime of an individual is rather novel [6]. However, it follows directly from Gene x Environment interactions (GxE) influencing the reaction norm. Along the developmental axis Gene x Age (GxA) and Individual x Age (IxA) interactions will then determine the expression of the developing reaction norm in interaction with the environments encountered by an individual (see [7]). As pointed out earlier [2] genes themselves provide an environment for other genes so that even more complex interactions are to be expected. As these interactions may change with the state and the age of an individual it becomes clear why ontogenetic processes are of prime relevance for understanding potential adaptive effects of plasticity.

Complex models of ontogenetic processes (mostly taken from quantitative genetics; like animal models) are needed to describe such interactions (see [7]) and thereby characterize the reaction norm's dependence on internal and external influences over the lifetime. We stress that these ontogenetic processes shape animal behaviour over the lifetime and - through trans-generational effects – even beyond. This view leads to several major questions which we have addressed in our 2014 workshop at the Center for Interdisciplinary Research (ZIF, Bielefeld, Germany) and treat to various depths here in our collection of papers:

1) why do we observe ontogenetic plasticity and how does it come about in the interaction of GxExA?

2) how do changes in the reaction norm and the observed plasticities influence the measurement of repeatabilities and correlations among different behaviours (and other traits) of the individual?

3) These processes involve substantial self-organisation (as observed even when iso-genetic individuals living in the same environment become differentiated; [8,9]).This self-organisation becomes perhaps particularly amenable to measurement during certain windows of time when individuals readjust their phenotypes. Can we predict when such time windows would be most adaptive with respect to critical life stages or conditions of the environment that provide cues with particularly high predictive power (like diurnal or annual changes of conditions)? Does such plasticity of the reaction norm shrink with age or disappear completely?

4) Lastly, can we understand the mechanisms behind the width and plasticity of reaction norms for different traits beyond the answers that theory and animal models can provide?

In the collection of papers in this special issue, Brommer & Class [7] explore the plasticity of behaviour in response to age. They make use of the theory of reaction norms to predict how age-related changes in behaviour may affect repeatability and hence heritability of behaviour. Furthermore, they elaborate how developmental plasticity can affect covariances with other behaviours. The importance of particular life stage for the uptake of environmental cues and adjustment in behaviours is further discussed by Fawcett & Frankenhuis [10]. They model how cue frequency, cue reliability and cue importance may differ during life stages based on the life history of the organism. Based on differences in environmental cues, the authors predict when selection processes should lead to the evolution of ‘sensitive windows’- periods during development in which organisms show increased susceptibility to (certain) environmental cues. Del Giudice [11] further explores the idea of plasticity as a developing trait that may be modulated by the environment an organism experiences. He presents a model that explicitly includes humans showing how cue reliability can affect the evolution of plasticity. Thereby a call of Fawcett & Frankenhuis [10] is answered who suggested that such a model should be formulated. In a further conceptual paper, Han & Dingemanse [12] focus on one of the most important environmental cues, the macronutrients contained in the diet, and suggest how diet composition may exert pleiotropic effects on personality and behavioural syndromes both, short term and over evolutionary time. Employing the same statistical framework as [7] to explain how GxA interaction may affect covariances between behaviours and repeatability/heritability, Han & Dingemanse show how GxE interactions may affect these properties. Groothuis & Taborski [13] then point out different mechanisms by which environmental influences may interact with sensitive windows to affect the organism's performance. They critically discuss frequently used experimental designs by behavioural ecologists and neurobiologists and highlight the importance of full, reciprocal designs for understanding the consequences of environmental conditions across development.

Behavioural changes are often believed to be mediated by hormonal changes. Hau & Goyman [14] review evidence for consistent behaviour-hormone relationships within and between individuals in birds. Focusing on two hormones that are well known to change predictably during the ontogeny of organisms (corticosterone and testosterone), they conclude that our understanding of hormone-behaviour relationships on an individual level is rather limited. They call for better characterisation of individual hormonal phenotypes, their responses to environmental change and covariation with behaviours by employing a reaction norm approach.

On the empirical side, Müller & Müller [15] and Würz & Krüger [16] explore how age related changes in behaviour affect repeatability of behaviour within and between distinct life stages in an insect and a bird species. As expected, both studies conclude that behaviours are more repeatable within life stages than between, but more surprisingly show that some behaviours only become repeatable when the individual reaches a certain life stage. While in the leaf beetle [15] correlations among behaviours are mostly stable even across life stages, this is not the case in zebra finches [16]. An empirical example for the importance of naturally occurring differences in early diet composition on the expression of behaviour later in life is given by Van Oers et al. [17]. Great tit chicks provided with fewer caterpillars during the first days after hatching exhibited a stronger stress response later in life. Krause & Naguib [18] demonstrate how early diet affects the development of sexual ornaments and behaviours later in life. Although birds can catch up in expression of sexual ornaments quickly, other traits, like body mass, exploration and activity are affected for much longer, revealing a possible trade-off in resource allocation to compensate for poor early conditions. Two more empirical studies investigate the development of personality traits in infants, a life history stage for which data on personality are extremely scarce, of three different mammalian species. Hudson et al. [19] investigate how locomotion behaviour and separation calls develop in cats and mound-building mice, showing that both behaviours are repeatable and may be used to enhance our understanding of personality during early ages in future studies. Von Engelhardt et al. [20] study how development from birth to independence is affected by maternal social environment during pregnancy. Comparable to the study by Hudson et al. [19], they show that locomotion behaviour and separation calls are not correlated but that separation calls one day after birth predict activity in an unknown environment later in life.

Trans-generational effects are the focus of the study of Kinnally & Capitanio [21]. Rhesus macaques reared in a nursery show stronger physiological stress responses and more emotional behaviour when their fathers were nursery reared compared to offspring of fathers that were reared by their mother. This paper demonstrates that the (adaptive?) shaping of behaviour does not end within the lifetime of an organism but extents even to future generations. Mechanisms that lead to differences in personality may also be involved in evolutionary processes (through processes like genetic assimilation). Kappeler & Fichtel [22] describe the ‘lemur-syndrome’, a set of behavioural, morphological and ecological traits unique to Madagascan lemurs. They hypothesise how trans-generational effects of chronic maternal stress due to adverse environmental conditions and developmental consequences may have led to evolutionary stable life history adjustments. In contrast, Oldfield et al. [23] suggest how a broadly influential underlying mechanism might have been modified during evolution permitting its re-use in different environmental circumstances across a wide array of vertebrate taxa.

Brust et al. [24] review literature on laboratory mice (accounting for more than 70% of animals used in biological studies with fundamental nature) development and point out that even for this extensively studied species knowledge is scarce about most life stages and in particular about the environmental cues potentially influencing development of the behavioural phenotype during ontogeny. In search for potential mechanisms, Hennessy et al. [25] review evidence of hormonal, immunological and behavioural plasticity in guinea pigs. They explore in which way social experiences shape the responsiveness of the hypothalamic-pituitary-adrenocortical axis and immune-related activity during different stages of life. Ultimately these processes lead to predictable behavioural changes suggesting lifelong adaptive shaping of the organism. Shaping of behaviours and hormones across multiple generations to adapt organisms to man-made living conditions is called domestication. Kaiser et al. [26] describe how domestication has reshaped physiological and behavioural profiles and their development during ontogeny in guinea pigs as compared to their wild congeners, the cavy. They show that domestication processes affected suites of traits thereby altering behavioural syndrome structure between the two forms and discuss the evolutionary significance of these differences.

While behavioural and evolutionary biologists have acknowledged variability within and between individuals as important, behavioural neuroscientists have aimed to reduce variability as much as possible for decades in so-called translational (applied and pre-clinical) research. Standardisation procedures encompass housing conditions, experimental procedures, species studied, sex, age and even genetic background. In their contribution, Macrì & Richter [27] warn against such practices, pointing out the potential pitfalls of reduced reproducibility of results. They also stress that such a narrow comparative view, neglecting the fact of behavioural plasticity will reduce the option to translate and apply such research to human clinical problems. In the last paper of this collection, Crews et al. [28] strongly remind us that old concepts, particularly on the norms of reaction and the concept of plasticity published decades ago should not be forgotten as otherwise we may reinvent the wheel or repeat old mistakes. By means of five examples they exemplify: The interaction of heredity and environment cannot be understood outside the context of development!

Overall, this impressive collection provides a broad view of the importance of the ontogeny of the reaction norm and of the potential for development of its plasticity during the lifetime, thereby stressing the pervasive importance of the study of ontogenetic processes.

## References

[B1] Stamps JA Groothuis TGG Developmental perspectives on personality: Implications for ecological and evolutionary studies of individual differences Philosophical Transactions of the Royal Society Series B 2010 365 1560 4029 4041 10.1098/rstb.2010.0218 PMC2992751 21078655

[B2] Groothuis TGG Trillmich F Unfolding personalities: The importance of studying ontogeny Developmental Psychobiology 2011 53 6 641 655 10.1002/dev.20574 21866544

[B3] Sachser N Kaiser S Hennessy MB Behavioural profiles are shaped by social experience: when, how and why Philosophical Transactions of the Royal Society Series B 2013 368 1618 20120344 10.1098/rstb.2012.0344 PMC3638447 23569292

[B4] Stamps JA Individual differences in behavioural plasticities Biol Rev Camb Philos Soc 2015 10.1111/brv.12186 25865135

[B5] Dall SR McNamara JM Leimar O Genes as cues: phenotypic integration of genetic and epigenetic information from a Darwinian perspective Trends in Ecology & Evolution 2015 30 6 327 333 10.1016/j.tree.2015.04.002 25944666

[B6] Pluess M Belsky J Prenatal programming of postnatal plasticity? Development and Psychopathology 2011 23 1 29 38 10.1017/S0954579410000623 21262037

[B7] Brommer JE Class B The importance of genotype-by-age interactions for the development of repeatable behavior and correlated behaviors over lifetime Frontiers in Zoology 2015 12 Suppl 1 S2 10.1186/1742-9994-12-S1-S2 PMC4722339 26816518

[B8] Lewejohann L Zipser B Sachser N “Personality” in laboratory mice used for biomedical research: A way of understanding variability? Developmental Psychobiology 2011 53 6 624 630 10.1002/dev.20553 21866543

[B9] Freund J Brandmaier AM Lewejohann L Kirste I Kritzler M Krüger A Emergence of individuality in genetically identical mice Science 2013 340 6133 756 759 10.1126/science.1235294 23661762

[B10] Fawcett RW Frankenhuis WE Adaptive explanations for sensitive windows in development Frontiers in Zoology 2015 12 Suppl 1 S3 10.1186/1742-9994-12-S1-S3 PMC4722342 26816521

[B11] Del Giudice M Plasticity as a developing trait: exploring the implications Frontiers in Zoology 2015 12 Suppl 1 S4 10.1186/1742-9994-12-S1-S4 PMC4722347 26816522

[B12] Han CS Dingemanse NJ Effect of diet on the structure of animal personality Frontiers in Zoology 2015 12 Suppl 1 S5 10.1186/1742-9994-12-S1-S5 PMC5385817 28400852

[B13] Groothuis TGG Taborsky B Introducing biological realism into the study of developmental plasticity in behaviour Frontiers in Zoology 2015 12 Suppl 1 S6 10.1186/1742-9994-12-S1-S6 PMC4722348 26816523

[B14] Hau M Goymann W Endocrine mechanisms, behavioral phenotypes and plasticity: known relationships and open questions Frontiers in Zoology 2015 12 Suppl 1 S7 10.1186/1742-9994-12-S1-S7 PMC4722346 26816524

[B15] Müller T Müller C Behavioural phenotypes over the lifetime of a holometabolous insect Frontiers in Zoology 2015 12 Suppl 1 S8 10.1186/1742-9994-12-S1-S8 PMC4722364 26816525

[B16] Würz Y Krüger O Personality over ontogeny in zebra finches: long-term repeatable traits but unstable behavioural syndromes Frontiers in Zoology 2015 12 Suppl 1 S9 10.1186/1742-9994-12-S1-S9 PMC4722341 26813709

[B17] van Oers K Kohn GM Hinde CA Naguib M Parental food provisioning is related to nestling stress response in wild great tit nestlings: implications for the development of personality Frontiers in Zoology 2015 12 Suppl 1 S10 10.1186/1742-9994-12-S1-S10 PMC4755007 26913051

[B18] Krause TE Naguib M Zebra finch males compensate in plumage ornaments at sexual maturation for a bad start in life Frontiers in Zoology 2015 12 Suppl 1 S11 10.1186/1742-9994-12-S1-S11 PMC4722338 26816511

[B19] Hudson R Rangassamy M Saldaña A Bánszegi O Rödel HG Stable individual differences in separation calls during early development in cats and mice Frontiers in Zoology 2015 12 Suppl 1 S12 10.1186/1742-9994-12-S1-S12 PMC4722366 26816512

[B20] von Engelhardt N Kowalski GJ Guenther A The maternal social environment shapes offspring growth, physiology, and behavioural phenotype in guinea pigs Frontiers in Zoology 2015 12 Suppl 1 S13 10.1186/1742-9994-12-S1-S13 PMC4722343 26816513

[B21] Kinnally EL Capitanio JP Paternal Early Experiences Influence Infant Development through Non-Social Mechanisms in Rhesus Macaques Frontiers in Zoology 2015 12 Suppl 1 S14 10.1186/1742-9994-12-S1-S14 PMC4722344 26816514

[B22] Kappeler PM Fichtel C Eco-evo-devo of the lemur syndrome: Did adaptive behavioral plasticity get canalized in a large primate radiation? Frontiers in Zoology 2015 12 Suppl 1 S15 10.1186/1742-9994-12-S1-S15 PMC4722368 26816515

[B23] Oldfield RG Harris RM Hofmann HA Integrating resource defence theory with a neural nonapeptide pathway to explain territory-based mating systems Frontiers in Zoology 2015 12 Suppl 1 S16 10.1186/1742-9994-12-S1-S16 PMC4722349 26813803

[B24] Brust V Schindler PM Lewejohann L Lifetime development of behavioural phenotype in the house mouse (*Mus musculus*) Frontiers in Zoology 2015 12 Suppl 1 S17 10.1186/1742-9994-12-S1-S17 PMC4722345 26816516

[B25] Hennessy MB Kaiser S Tiedtke T Sachser N Stability and change: Stress responses and the shaping of behavioral phenotypes over the life span Frontiers in Zoology 2015 12 Suppl 1 S18 10.1186/1742-9994-12-S1-S18 PMC4722350 26816517

[B26] Kaiser S Hennessy MB Sachser N Domestication affects the structure, development and stability of biobehavioural profiles Frontiers in Zoology 2015 12 Suppl 1 S19 10.1186/1742-9994-12-S1-S19 PMC5385816 28400853

[B27] Macrì S Richter H The Snark was a Boojum – reloaded Frontiers in Zoology 2015 12 Suppl 1 S20 10.1186/1742-9994-12-S1-S20 PMC4722367 26816519

[B28] Crews D Weisberg SA Sahotra S Sarkar Hazards Inherent in Interdisciplinary Behavioral Research Frontiers in Zoology 2015 12 Suppl 1 S21 10.1186/1742-9994-12-S1-S21 PMC4722337 26816520

